# The Sewage Farms of Berlin

**Published:** 1891-01

**Authors:** 


### THE SEWAGE FARMS OF BERLIN.

The meeting of the International Medical Congress at Berlin in the first week of August afforded to the members the advantage of being conducted over the famous sewage farms connected with that city. Berlin is divided into twelve districts, each of which has its own pumping station, which sends out the sewage of its part of the one and a-half million of inhabitants composing the population of Berlin to the different farms purchased by the municipality. The total extent of the farms used for the purification of the sewage is about 19,000 acres. The farms are situated partly on the north and partly on the south of the city. The southern ones are most beautiful and successful farms, and about 71 per cent. of the ground is irrigated by the sewage; 96 per cent. of this irrigated part is drained. The length of the pipes which convey the sewage to the farms varies from about five-eighths of a mile to about 11½ miles, and the diameter of the main tubes varies from one meter to three-quarters of a meter. Once arrived at the farms the diameter of the pipes is lessened, and, finally, those used to convey the sewage to the fields do not exceed one-fifth of a meter. The conduits end at the highest point of the ground to be irrigated, and the most inclined fields are employed as meadows, whilst the flatter fields are used for the cultivation of roots; and some fields are covered in winter time by sewage for some months, and then used for the production of cereals in spring. The water is conveyed from the highest point of the fields by ditches, half a meter in depth, and where root crops are concerned the sewage is allowed only to touch the roots of the plants; but in the case of meadows it flows over the whole surface of the meadow. The idea that such farms become unfit for use in some years by clogging up, and then unable to purify sewage any longer, is now known to be erroneous, and after many years of use the sewage water is still only a slightly muddy fluid, and the effluent is pure, clear, and inodorous. The city of Berlin has taken the advantage of the existence of these farms to employ a number of persons in agriculture. Some of the workmen receive in wages and kind a value of about $300 yearly. The day laborers receive about $100, and women about $50, with lodging and farm produce, which makes the yearly income of each family about $300 also. There are also about 900 paupers who are employed on the farms, according to their powers. The produce of their labor is estimated as worth about one-fourth of that of the ordinary laborers. The health of the population employed on the farms has been examined by Professor Virchow, and found to be excellent. Eventually, I feel sure that all cities will imitate Berlin.

---

East Springfield, Erie Co., Penn., Dec. 7, 1890.

Herba Vita Remedy Co.

The Herba Vita I got of you some time ago is all gone and I would like more. The small packages you sent me I distributed and probably you have heard from some of them. I do not like to be without it in my family. Send 1 doz. $1 packages by express via Nickel Plate R. R., and I will remit on receipt of bill.

Respectfully yours,

J. M. STRONG.

